# The Effect of the Low-Carbon City Pilot Program on the Cognitive Function of Older Adults: Quasi-Experimental Evidence from China

**DOI:** 10.1093/geront/gnaf131

**Published:** 2025-04-17

**Authors:** Jin Ke, Fei Sun

**Affiliations:** Elderly Service Research Center, School of Sociology, Huazhong University of Science and Technology, Wuhan, China; School of Social Work, Michigan State University, East Lansing, Michigan, USA

**Keywords:** Chinese older adults, Cognitive function, Low-carbon policy

## Abstract

**Background and Objectives:**

In response to the impact of climate change, China initiated the Low-Carbon City Pilot (LCCP) program in 2010. Despite the program’s positive environmental outcomes, its influence on health among older adults—an age group highly vulnerable to air pollution—remains understudied. This study aims to investigate the effect of the LCCP program on cognitive health among older Chinese and explore the intermediate pathways involved.

**Research Design and Methods:**

Utilizing data from the China Health and Retirement Longitudinal Study (CHARLS), this research employs a staggered difference-in-differences estimator to examine the effects of the LCCP program on cognitive health of older Chinese.

**Results:**

The findings indicate that the LCCP program has been associated with relative improvements in cognitive function of older adults in China, and that this observed positive association has increased over time. Mechanism analysis identifies the enhanced outdoor and indoor air quality, the expansion of green spaces in the city, and increased individual outdoor activity as possible channels through which the LCCP program has exerted its beneficial effects.

**Discussion and Implications:**

The positive association of the LCCP program with cognitive function in Chinese older adults highlights the interconnected nature of environmental and health outcomes. As cities worldwide grapple with the challenges of an aging population and climate change, the insights from this study offer practice implications for designing and refining low carbon city initiatives.

Air pollution, significantly affected by China’s rapid economic growth, has emerged as a major environmental concern (Yang et al., 2020). Air pollution has posed health risks for Chinese residents, leading to premature mortality for approximately 350,000–500,000 Chinese urban dwellers annually due to high levels of particulate matter in the air (Chen et al., 2013). Older adults are particularly vulnerable to the adverse effects of environmental pollution due to age-related physiological changes and the presence of common comorbidities such as cardiovascular disease, chronic respiratory conditions (e.g., COPD), and diabetes. Environmental stressors, including air pollution, may exacerbate the underlying aging process through mechanisms such as oxidative stress and systemic inflammation, both of which are implicated in multiple chronic diseases. Furthermore, air pollution has been identified as one of the 14 modifiable risk factors for Alzheimer’s disease and related dementias (Livingston et al., 2020, 2024) to which older adults are especially susceptible.

The issue of air pollution is especially critical for the aging population in China, given the country’s significant increase in its older population. In 2010, China had 177 million individuals aged 60 or above, representing 13.6% of its total population. This number grew to 267 million by 2021, accounting for 18.9% of the total population (Du, 2023). This demographic highlights a critical need for the government’s effort to reduce air pollution to protect and improve the health and well-being of the aging population (Wang et al., 2019).

In response to this challenge, the Chinese government initiated the Low-Carbon City Pilot (LCCP) program in 2010 to reduce air pollution and improve the health and well-being of residents. This initiative mirrors similar strategies in developed nations like Germany and France, where low-carbon zones have been established (Margaryan, 2021). While the existing literature has examined the health impact of low-carbon cities on older adults, most studies originated from developed countries and focused on the impact on the physical health (Margaryan, 2021; Poulhès & Proulhac, 2021). Recent research on the Chinese LCCP program has examined its effects on resident physical and mental health (Mu et al., 2022; Zhang et al., 2024). However, the influence of the low-carbon policies on older adults’ cognitive function is understudied. Preserving cognitive function of older adults is an essential part of active and healthy aging, and air pollution has been associated with accelerated cognitive decline in older adults (Bauman et al., 2016; Zhang et al., 2018).

Furthermore, as a developing country, China grapples with a legal system that is not as well established as those in developed countries. This situation often presents formidable challenges to the successful implementation of regional environmental policies, necessitating considerable public support to achieve the desired environmental regulatory outcomes (Li et al., 2016). A thorough assessment of the effect of the LCCP program on the cognitive function of older adults in the Chinese context is urgently needed.

## Development of the LCCP in China

The accelerated economic development of Chinese cities has been coupled with annual increases in energy consumption, air pollution, and greenhouse gas emissions (Sarkodie & Strezov, 2019). In a concerted effort to address these environmental issues, the Chinese government has implemented three successive phases of LCCP programs between 2010 and 2017. The first wave of pilots launched in 2010 included five provinces and eight cities in other provinces. Subsequently, the second wave was launched in 2012 and included Hainan Province and 28 other cities. Based on the experience of the previous two phases of LCCP programs, the Chinese government further expanded the program’s reach in 2017 and rolled out the third wave of LCCP programs in 45 cities and counties (see Appendix Figure for the specific distribution of the three waves of LCCP programs).

Pilot cites within the program are tasked with exploring a low-carbon green development model appropriate for the region that is in harmony with local natural conditions, resource availability, and economic foundation. Specific policies typically encompass three components. First, pilot cities adjust industrial structure to develop green and low-carbon industries to reduce air pollutants and CO_2_ emissions. Concurrently, there is an emphasis on technological upgrades within traditional industries to achieve greener and low-carbon transformation. Second, pilot cities call for a reduction in the use of certain fossil fuels (e.g., coal), energy efficiency in building design, and clean energy supply infrastructure such as natural gas pipelines. Third, pilot cities expand green spaces and develop public transportation networks. These measures are designed to simultaneously reduce air pollution and carbon emissions while offering residents more sustainable and eco-friendly commuting options. This multifaceted approach used in LCCP underscores the commitment to creating cleaner, healthier urban environments while fostering sustainable development practices.

## Environment Risks for Cognitive Function and the Role of LCCP

**Figure 1.**
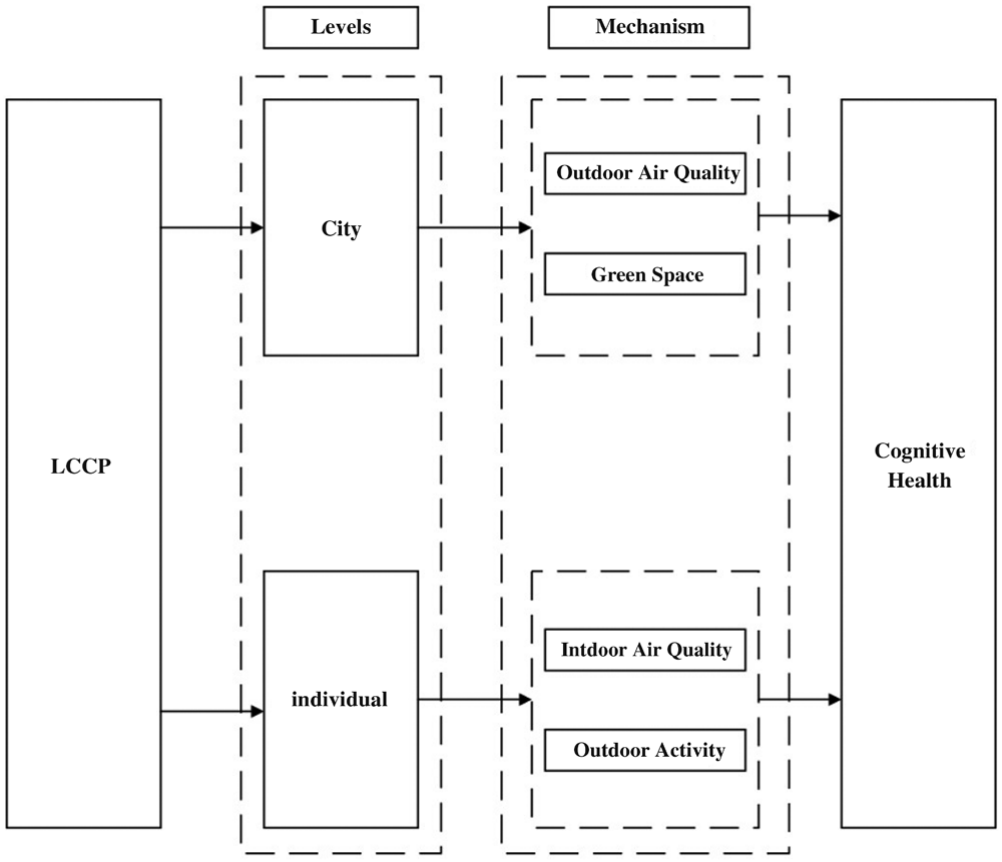
The conceptual framework for the relations among studied variables. LCCP = Low-carbon city pilot.

Given the established environmental risks to cognitive function, particularly from air pollution (Ailshire & Crimmins, 2014), a structured approach is needed to examine how policy interventions might mitigate these threats. Accordingly, this study utilizes the Environmental Risk Reduction Framework (Jones, 2001) to conceptualize the role of China’s LCCP Program in addressing these risks among older adults. This framework helps examine the role of macrolevel policy interventions in mitigating cognitive risks associated with environmental exposures. Figure 1 presents this conceptual model visually.

A primary environmental risk factor for cognitive health is air pollution. There is substantial empirical evidence linking long-term exposure to air pollution, particularly fine particulate matter (PM2.5), with adverse cognitive outcomes and accelerated cognitive decline in older adults (Zhang et al., 2018). Air pollution functions as a chronic environmental stressor that affects cognitive health through multiple pathways, including direct physiological harm (e.g., systemic inflammation, oxidative stress, and damage to neurons and brain structures essential for cognition) and indirect pathways via psychological stress and social vulnerabilities (Block & Calderón-Garcidueñas, 2009; Clifford et al., 2016). Furthermore, polluted environments may contribute to cognitive decline by limiting opportunities for health-promoting behaviors or exacerbating other modifiable risk factors such as physical inactivity, depression, and social isolation (Evans & Kantrowitz, 2002; Kulick et al., 2020). Recognizing these risks, the 2020 Lancet Commission on Dementia Prevention, Intervention, and Care identified air pollution as a key modifiable risk factor for dementia (Livingston et al., 2020).

Building on the Environmental Risk Reduction Framework, we posit that policy-level interventions like the LCCP can mitigate these cognitive risks. LCCP aims to reduce environmental stressors at the city level, while simultaneously promoting healthier lifestyle behaviors at the individual level. Specifically, LCCP may exert its protective effects through several key pathways.

One major pathway involves improving both outdoor and indoor air quality. LCCP policies directly target the reduction of outdoor air pollutants like PM2.5 through industrial adjustments and cleaner energy infrastructure (Chen et al., 2023; Zhang et al., 2022). Importantly, air pollution exposure also occurs indoors. In many homes, a major source is the use of solid fuels for cooking and heating. LCCP’s promotion of clean energy supply and technologies can facilitate a shift away from these polluting fuels (Qu et al., 2023), thereby improving indoor air quality, which is conducive to better cognitive function (Njenga et al., 2016; Saenz et al., 2021).

Increased access to green spaces represents another important protective pathway. Growing evidence indicates that exposure to green spaces can positively influence cognitive function in older adults (Chen et al., 2022). LCCP programs often include investments in green infrastructure, expanding urban green spaces. This expansion potentially enhances cognitive benefits (Besser, 2021).

Finally, LCCP can influence outdoor activity levels. Environmental conditions significantly shape lifestyle behaviors like physical activity. Poor outdoor air quality can deter individuals from spending time outdoors (Hu et al., 2017), while engaging in such activities is known to support cognitive health (Jung et al., 2018; Leyland et al., 2019). Improvements in the external environment resulting from the LCCP may encourage older adults to engage in more outdoor activities, a behavioral change that could be related to enhanced cognitive function.

## Research Aims and Hypotheses

In line with the evidence above, we hypothesize that older Chinese in cities with LCCP would have better cognitive function (Hypothesis 1) than their counterparts living elsewhere. At the city level, the LCCP program can contribute to improved external living environments such as better air quality and greater availability of green spaces, which leads to beneficial impact on cognitive function. At the individual level, the LCCP program would help improve domestic indoor air quality and increase engagement in outdoor activities. Specific hypotheses regarding the four possible mechanisms are described below.

**Hypothesis 2a** proposes that the LCCP program would contribute to cognitive function of older adults by improving outdoor air quality. The concentration of PM2.5 was used as an indicator for outdoor air quality. We hypothesize that LCCP would reduce the PM2.5 exposure in older adults, leading to improved cognitive function. **Hypothesis 2b** assumes that the LCCP program would help improve cognitive function of older adults by increasing their exposure to urban green space. **Hypothesis 2c** proposes that the LCCP program would improve the cognitive function of older adults by improved indoor air quality. **Hypothesis 2d** suggests that the LCCP program can improve cognitive function of older adults by increasing their engagement in outdoor activities. Figure 1 depicts the relationship among studied variables.

## Method

This secondary data analysis study used data from the China Health and Retirement Longitudinal Survey (CHARLS) database, a large national representative database from Peking University (Zhao et al., 2014). The CHARLS encompassed survey data from individuals and households aged 45 years and older in China, offering information on demographic characteristics, health status, economic status, and healthcare utilization. The data covered 150 counties and 450 communities (villages) in 28 provinces in China, with a sample of 12,400 households and approximately 19,000 individuals at each wave. The first survey of the CHARLS program was conducted in 2011, and follow-up surveys were conducted in 2013, 2015, and 2018. The CHARLS database has been widely used to study the health status, long-term care needs, and healthcare utilization of older adults (Zhao et al., 2020).

Specifically, four waves of CHARLS data from 2011, 2013, 2015, and 2018, were used to examine the cognitive function changes associated with the implementation of the LCCP program using the difference-in-difference (DID) strategy. We selected individuals aged 60 or above for defining an older adult, mainly because in mainland China the prevalent retirement age is 60, the point at which individuals start to receive social security benefits. After excluding participants under age 60, we obtained a four-wave panel dataset consisting of 28,497 older adults aged 60 years and older from 123 cities (6,304 in 2011, 6,965 in 2013, 7,668 in 2015, and 7,560 in 2018). Missing data was dealt with listwise deletion approach.

Furthermore, we retrieved climate-related data from the Atmospheric Composition Analysis Group of Dalhousie University and city characteristics from the China City Statistical Yearbook 2009–2020. We created a panel dataset of 282 cities including economic, environmental, and climate variables for the periods between 2009 and 2020. For further details, please refer to Panel B in Table 1.

**Table 1.** Descriptive Statistics of Individual and City Level Panel Variables

Variables	*SD*	Min	Median	Max	Mean			
					Total	Control	Treated	Difference
***Panel A Sample descriptive statistics for the main regression***								
***Dependent variable***								
MMSE	5.847	0.000	12.000	21.000	11.676	11.600	11.830	−0.234^***^
***Independent variables***								
***Individual-level characteristics***								
Age	6.736	60.000	67.000	108.000	68.484	68.520	68.400	0.122
Male (yes = 1; no = 0)	0.500	0.000	0.000	1.000	0.487	0.484	0.493	−0.008
Agricultural Hukou (yes = 1; no = 0)	0.404	0.000	1.000	1.000	0.795	0.814	0.757	0.057^***^
Married (yes = 1; no = 0)	0.408	0.000	1.000	1.000	0.789	0.790	0.789	0.001
Level of education								
Elementary School or below (control group)	0.380	0.000	1.000	1.000	0.825	0.833	0.809	0.024^***^
Middle school	0.322	0.000	0.000	1.000	0.117	0.113	0.126	−0.013^***^
High school	0.209	0.000	0.000	1.000	0.046	0.043	0.052	−0.009^***^
University or above	0.108	0.000	0.000	1.000	0.012	0.011	0.013	−0.002
Smoke (yes = 1; no = 0)	0.474	0.000	0.000	1.000	0.342	0.344	0.337	0.008
Drink (yes = 1; no = 0)	0.462	0.000	0.000	1.000	0.309	0.309	0.307	0.003
Exercise (yes = 1; no = 0)	0.012	0.000	1.000	1.000	1.000	1.000	1.000	0.000
PHI (yes = 1; no = 0)	0.249	0.000	1.000	1.000	0.933	0.934	0.931	0.003
ADL	1.161	0.000	0.000	6.000	0.540	0.550	0.520	0.030**
CES-D score	6.450	0.000	8.000	30.000	8.915	8.896	8.953	−0.058
***Household-level characteristics***								
Running water (yes = 1; no = 0)	0.455	0.000	1.000	1.000	0.707	0.672	0.777	−0.104^***^
Toilet flushable (yes = 1; no = 0)	0.474	0.000	0.000	1.000	0.341	0.314	0.395	−0.081^***^
Logarithm of household income per capita	3.921	0.000	7.548	18.422	6.411	6.288	6.660	−0.372^***^
Number of family members	1.657	1.000	2.000	16.000	2.855	2.824	2.919	−0.095^***^
Obs.	28,497				28,497	19,039	9,458	
***Panel B Statistics for city-level variables***								
**Air quality**								
PM 2.5 (log)	0.369	3.693	1.150	4.687	3.698	3.745	3.596	0.150^***^
**Green spaces**								
Green space area (million hectares)	0.286	0.084	0.008	1.999	0.172	0.124	0.275	−0.151^***^
***Weather variables***								
Wind speed (m/s)	1.012	4.737	2.767	7.533	4.788	4.754	4.862	−0.108^***^
Rainfall (mm)	13.046	25.477	7.463	61.592	28.163	26.560	31.640	−5.080^***^
Temperature (degree Celsius)	5.094	15.608	3.022	24.018	14.625	13.980	16.040	−2.062^***^
***City Characteristics***								
GDP per capita (yuan; log)	0.715	10.545	9.085	12.454	10.615	10.500	10.870	−0.372^***^
Industrial wastewater emissions (tons; log)	1.130	8.284	4.868	10.590	8.178	8.151	8.237	−0.086**
Sulfur dioxide emissions per unit area (tons/km^2^)	10.745	2.609	0.046	63.308	6.685	6.818	6.396	0.422
Smoke and dust emissions per unit area (tons/km^2^)	3.543	1.298	0.035	20.116	2.595	2.663	2.448	0.215
Centralized disposal rate of urban sewage (%)	15.977	89.770	25.270	100.000	84.130	82.250	88.210	−5.953^***^
GDP share of primary sector (%)	7.889	12.020	0.660	39.460	12.835	12.980	12.520	0.455
GDP share of secondary sector (%)	10.914	47.000	16.290	71.770	46.208	46.590	45.380	1.206^***^
GDP share of tertiary sector (%)	9.717	40.060	20.680	69.180	40.925	40.410	42.050	−1.641^***^
Percentage of unemployed population (%)	3.161	5.340	0.965	19.234	5.891	6.075	5.490	0.585^***^
Passenger traffic by road (tens of thousands; log)	1.044	8.355	5.517	11.266	8.356	8.324	8.425	−0.102^***^
Obs.	3,384				3,384	2,318	1,066	

*Note*: In Panel A, individuals covered by LCCP (treated) and individuals not covered by LCCP (control) were compared using *t* tests. In Panel B, cities covered by LCCP (treated) and cities not covered by LCCP (control) were compared using *t* tests. ADL = activities of daily living; CES-D = Center for Epidemiological Studies Depression; GDP = gross domestic product; LCCP = Low-carbon city pilot; MMSE = Mini Mental State Examination; PHI = public health insurance; PM = particulate matter; *SD* = standard deviation.

***p < .01. **p < .05.

*p < .1.

### Measurement

#### Dependent variable

The Chinese version of the Mini-Mental Status Examination (MMSE) was used to measure cognitive function. The validity and reliability of the Chinese version of MMSE have been confirmed in previous studies (Jia et al., 2021). This Chinese version of MMSE with a total score of 21 assessed episodic memory, orientation, attention and calculation, and visual-spatial skills, and has been used validated in numerous studies (e.g., Qin et al., 2019; Yu et al., 2022). The summed score of all the sections ranged from 0 to 21, with higher scores indicating better cognitive function.

#### Independent variable

The independent variable, LCCP was a binary variable assigned a value of 1 to individuals covered by the LCCP program in the survey year and 0 otherwise. As the LCCP programs were piloted at three different times in various cities, the values of LCCP were defined accordingly.

### Hypothesized Mediators

City-level mediators consisted of outdoor air quality assessed by PM2.5 concentrations, and green spaces per capita by the area of green spaces. Individual-level meditators included in-door air quality and outdoor activity. Referring to existing studies, the use of clean cooking fuels or not is a good proxy for indoor air quality (Halder et al., 2024), and the use of clean cooking fuels tends to imply better indoor air quality (Imelda, 2020). Therefore, we used the use of clean cooking fuels as a proxy variable for indoor air quality, while outdoor activity was indicated by the average monthly frequency of such activity.

### Control Variables

We controlled variables critical to cognitive function in our first hypothesis testing. Referring to existing work (Krivanek et al., 2021; Mortensen et al., 2014) on cognitive function, we controlled for individual and household-level characteristics. Individual-level characteristics included basic demographic characteristics such as age, gender, level of education, marital status, registration type (*HuKou*), health insurance coverage, and health behaviors such as engagement in smoking, drinking, or exercising. Furthermore, we controlled for the individual’s physical health and mental health as measured by the activity of daily living scale and the CES-D (Center for Epidemiologic Studies Depression) scale, where activity of daily living status was measured by the amount of help the individual needed in bathing, dressing, eating, getting in/out of bed, using the toilet, and controlling urination (Yang et al., 2024), while the CES-D scale has a total score of 30, with higher scores indicating higher levels of depression (Yan et al., 2024). Household-level characteristics include whether the house has running water, whether the house has a flushing toilet, the annual per capita household income, and the number of household members. In our mediation analyses, we incorporated city-level covariates, including weather variables such as wind speed, rainfall, and temperature, economic indicators like GDP per capita and environmental factors, such as wastewater emissions and sulfur dioxide emissions.

### Analytic Strategies

We adopted the heterogeneity robust DID estimator proposed by de Chaisemartin and D’Haultfœuille (2020) to test the effect of LCCP on cognitive function, addressing our first hypothesis. This approach overcomes the problem of possible negative weights inherent in the two-way fixed effects model that is commonly used in previous research for staggered DID estimation. Specifically, if there is heterogeneity in the average treatment effects across time or groups, the treated observations may be incorrectly considered as the control group. Due to the possible negative weights, the average treatment effects can be positive but the linear regression coefficient may be negative (de Chaisemartin & D’Haultfœuille, 2020). Consequently, the use of the heterogeneity robust DID estimator to assess the effect of the LCCP program on the cognitive function of older adults will lead to more precision of estimated causal effects. To illustrate, in this approach, observations are divided into I groups and T time periods, respectively denoted as i∈{1,...,I} and t∈{1,...,T}. The estimation process is specified as follows:


$$\begin{array}{l} DID_{i,l}=E\left[Y_{i,F_{i}+l}(0_{F_{i}-1},1_{l+1})-Y_{i,F_{i}+l}(0_{F_{i}+l})\right] \end{array}$$

where $F_{i}$ denotes the first period in which individual i is covered by the LCCP. l denotes the length of coverage by the LCCP. $Y_{i,F_{i}+l}$ denotes the cognitive function status of individual i in period $F_{i}+l$ as measured by the MMSE. $0_{F_{i}-1}$ denotes a $1\times (F_{i}-1)$ vector with coordinates equal to 0. Similarly, $0_{F_{i}+l}$ denotes a $1\times (F_{i}+1)$ vector with coordinates equal to 0. $1_{l+1}$ denotes a 1×(l+1) vector with coordinates equal to 1. In summary, $Y_{i,F_{i}+l}(0_{F_{i}-1},1_{l+1})$ represents the actual cognitive function status of individual i in the treatment group in period $F_{i}+l$. $Y_{i,F_{i}+l}(0_{F_{i}+l})$ denotes the counterfactual cognitive function “status quo” if an individual is not covered by the LCCP from the first period to period $F_{i}+l$. $DID_{i,l}$ is then the expected difference between $Y_{i,F_{i}+l}(0_{F_{i}-1},1_{l+1})$ and $Y_{i,F_{i}+l}(0_{F_{i}+l})$. Furthermore, following the recommendations of de Chaisemartin and D’Haultfœuille (2020), this paper clusters standard errors to the city level.

Due to the possible endogeneity of the mediating variable itself, which may be mutually causal with the dependent variable, the traditional stepwise approach to testing mediation effects could introduce estimation bias (Judd & Kenny, 1981) and fail to obtain consistent estimates (Angrist & Pischke, 2009). Therefore, we adopted Dell (2010)’s channel test framework to test the hypothesized mechanism. This framework focuses on the causal relationship between the independent variables and both the dependent and mediating variables. If the causal relationship of the mediating variable to the dependent variable is theoretically straightforward, obvious, and confirmed by a considerable body of research, formal causal inference techniques to investigate the causal relationship of the mediating variable to the dependent variable are considered unnecessary. This framework has been widely recognized and used in health policy research (e.g., Chen et al., 2020).

## Results

### Sample Characteristics

Descriptive statistics of the participants in the entire sample, including treated and control groups are presented in panel A of Table 1. The average age of the participants was 68.48 years (standard deviation, *SD* = 6.74) and the median age was 67 years. *t* Tests indicated the level of significance for comparisons between the treated and control groups.

### The Effect of LCCP on Cognitive Function

Table 2 shows the effect of the LCCP program on the cognitive function of older adults. Panel A focuses on the average total effect of the LCCP program and shows that the LCCP program has significantly increased the MMSE score of the older population by 0.79 points.

**Table 2.** The Effect of LCCP Program on the Cognitive Function of Older Chinese

*Panel A* Estimation of treatment effects: Average total effect per treatment unit					
	Estimate	*SE*	95% CI	*N*	Switchers
Average effect	0.793	0.326	[0.155–1.431]	10,608	1,126
***Panel B*** Estimation of treatment effects: event-study effects					
	Estimate	*SE*	95% CI	*N*	Switchers
Effect_0	0.472	0.279	[-0.074 to 1.018]	6,977	684
Effect_1	1.116	0.456	[0.222–2.010]	3,208	263
Effect_2	1.546	0.534	[0.499–2.594]	2,226	179
***Panel C*** Testing the parallel trends and no anticipation assumptions					
	Estimate	*SE*	95% CI	*N*	Switchers
Placebo_1	−0.147	0.257	[−0.651 to 0.357]	2,651	255

*Notes*: CI = confidence interval; LCCP = xxx; SE = standard error. Individual fixed effect, time fixed effect, and covariates have been automatically controlled for in the estimator. Robust standard errors are clustered at the city level. *N* denotes the actual sample size used in the regression. Switchers indicate the group that experienced a change in treatment during the study period. The average effect is the mean of the summed effects of the treatment increment at the time of implementation and in subsequent periods.

**Figure 2.**
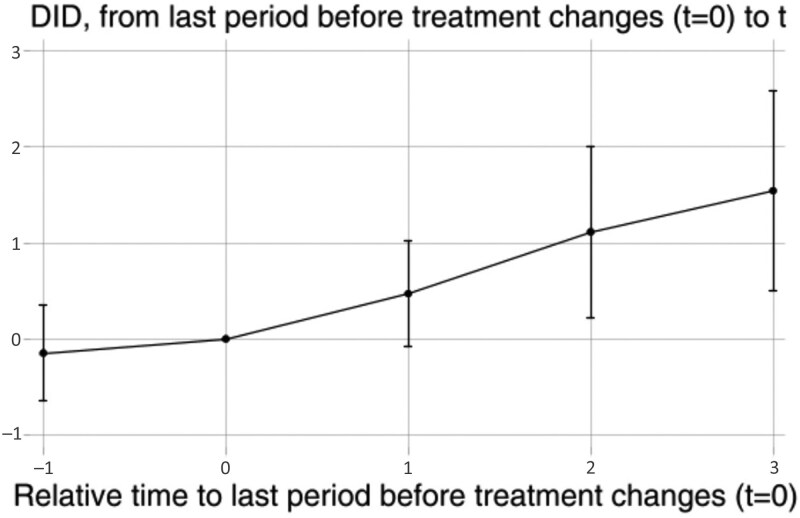
Dynamic and placebo effects of LCCP program on the cognitive function of older Chinese. DID = Difference in differences; LCCP = Low-carbon city pilot.

Panel B reports the results of decomposing the average effect into immediate and long-term effects, revealing that the positive association of the LCCP program on cognitive function is not immediate, but increases gradually over time. Those participants living in the areas covered by LCCP had better cognitive function than their counterparts who lived in areas not covered by LCCP. Additionally, the benefits on cognitive function appeared to strengthen with the duration of the program’s implementation. The lag effect can be attributed to two factors: a time lag in the implementation of the LCCP program, and the time needed to show improvement of cognitive function (Yuan & Grühn, 2021). Furthermore, the result of placebo test in Panel C affirmed that the DID strategy met the parallel trend assumption, indicating an equivalence between the treated and control groups prior to policy implementation. The dynamic and placebo effects are further depicted in Figure 2. We also conducted a robustness test using an alternative method by Callaway and Sant’Anna (2021) for solving the negative weights problem in staggered DID design. The robust test results confirmed our results here (data not shown).

### Results of Channels via Which LCCP Affects Cognitive Function

Table 3 reports the mechanism test results for the four channels of LCCP programs. At the city level, the LCCP programs resulted in a 2.3% [95% confidence intervals, CI −0.044, −0.001] reduction in PM2.5 concentrations and a 0.05 million hectare [95% CI 0.012, 0.088] increase of green spaces in the city. This means that the LCCP program has improved outdoor air quality and increased the likelihood of older adults having access to green space. At the individual level, the LCCP program positively increased the likelihood that their household would use clean cooking fuels by 4.4% [95% CI 0.008, 0.080] and increased their monthly frequency of outdoor activities by an average of 0.084 [95% CI 0.013, 0.155]. This means that the LCCP program has improved indoor air quality and increased the frequency of outdoor activities for older adults. Improvement in these four factors has consistently been shown to be a beneficial correlate of cognitive function (Besser, 2021; Leyland et al., 2019; Saenz et al., 2021). In line with the earlier mentioned channel test framework of Dell (2010), this suggests that LCCP programs may be positively associated with cognitive function through these mechanisms.

**Table 3.** Mechanisms of the Effect of LCCP Program on the Cognitive Function of Older Chinese

	City Level		Individual Level	
	Outdoor air quality	Green spaces	Indoor air quality	Outdoor activity
	Estimate [95% CI]	Estimate [95% CI]	Estimate [95% CI]	Estimate [95% CI]
Average Effect	−0.023 [−0.044 to −0.001]	0.050 [0.012 to 0.088]	0.044 [0.008 to 0.080]	0.084 [0.013 to 0.155]
Covariates	Y	Y	Y	Y
City/individual FE	Y	Y	Y	Y
Year effect	Y	Y	Y	Y
*N*	3,032	3,032	10,414	4,233
Switchers	996	996	1,112	410

*Notes*: CI = confidence interval; FE = fixed effect. Robust standard errors are clustered at the city level. The average effect is the mean of the summed effects of the treatment increment at the time of implementation and in subsequent periods. *N* denotes the actual sample size used in the regression. Switchers indicate the group that experienced a change in treatment during the study period.

## Discussion

This study examined the effect of the LCCP program on the cognitive function of Chinese older adults. The LCCP program was initiated in China as a pilot policy in response to the environmental concern associated with climate change. Prior research has documented environmental risk factors, in particular air pollution, for cognitive impairment or dementia (Livingston et al., 2020). The identified positive effect of LCCP on the cognitive function of older adults offers promising evidence for policy efforts to address environmental issues. Furthermore, this study used the staggered DID estimator, an approach better equipped to address the variability in policy implementation than the conventional two-way fixed effects approach, rendering this conclusion more accurate and less prone to bias.

Our main finding suggests that the LCCP program has been associated with relative improvements in cognitive function of older adults in China, and that this observed positive association has increased over time. This underscores the potential of public policies targeting environmental risk factors to directly influence cognitive function. This finding broadens the scope of existing research (Margaryan, 2021), which predominantly examined the effect of low-emission zones on physical health outcomes in developed countries. Our findings contributed new insights into the effects of a broader environmental initiative (i.e., LCCP) involving low-emission zones, regarding the cognitive function of older adults, a group especially vulnerable to the adverse effects of air pollution (Livingston et al., 2024). The validity of our findings is further reinforced by the robustness test employing the heterogeneous robust DID estimator suggested by Callaway and Sant’Anna (2021), enhancing the reliability of our results.

Furthermore, our exploration of secondary hypotheses regarding the four mechanisms through which the LCCP program may exert its beneficial effects has been affirmed. Our findings supported four possible hypothesized mechanisms: improved outdoor air quality, expanded urban green spaces, improved indoor air quality, and increased frequency of individuals’ outdoor activity. These findings were consistent with previous literature (Njenga et al., 2016; Zhang et al., 2022), emphasizing the multifaceted benefits of such environmental policies. The positive effect of the LCCP on outdoor air quality can be inspirational for policymaking in the field of aging, as improving outdoor air quality in areas with aging populations can be a critical strategy for mitigating age-related cognitive decline (Ailshire & Crimmins, 2014). This also underscores the potential for further refinement of the LCCP program and other aging policies in China, addressing intersecting demands of aging and environmental sustainability.

Over the past two decades, the establishment of low-carbon cities/zones has become a prevalent and significant approach in developed nations to combat air pollution and enhance the overall well-being of their residents. It is noteworthy that, compared to developed countries, developing nations like China often contend with underdeveloped legal frameworks and encounter formidable challenges in implementing territorial environmental policies. The findings of our study indicate that the LCCP program, as a regional environmental policy, has shown effectiveness in China, one of the world’s most populous countries. Consequently, such policy initiatives can be considered a viable means to address air pollution and bolster the cognitive function of older adults. These insights offer potential solutions to address the issues of air pollution and aging societies that challenge many other developing nations.

Several limitations should be noted. First, our reliance on the MMSE as the sole measure of cognitive function provides an assessment of global cognition but lacks multidimensional detail; this reflects the practical constraints common to large-scale surveys like CHARLS. Second, the nonexperimental design means definitive causal conclusions cannot be drawn. However, we employed a robust quasi-experimental DID design, controlling for confounders and finding empirical support for the key parallel trends assumption, which strengthens the basis for inferring an association between the LCCP and cognitive function trajectories. Furthermore, while this paper delved into the effect of the LCCP program on cognitive function, there remains much room to explore the effect of the LCCP program on other health outcomes, including mental health wellbeing. Finally, due to data restrictions, we were not able to obtain outdoor and indoor air quality data from individuals’ actual living environments, which prevented more precise estimates.

In conclusion, by employing a heterogeneous robust DID estimator, this study has found the positive association of the LCCP program on the cognitive function of China’s older population. This result has offered crucial guidance for Chinese policymakers by shedding light on effective strategies for implementing, enhancing, and assessing the LCCP program. Furthermore, this study not only enriches the existing body of knowledge but also serves as a blueprint for other developing nations grappling with air pollution challenges amidst a growing aging population. These countries may consider adopting or tailoring the LCCP model to their unique contexts, potentially catalyzing global efforts to improve air quality and healthy aging.

## Supplementary Material

gnaf131_suppl_Supplementary_Material

## Data Availability

Data and analytic methods or materials are available for replication purposes upon the request sent to the corresponding author.
