# Supplementary material for: The Effect of the Low-Carbon City Pilot Program on the Cognitive Function of Older Adults: Quasi-Experimental Evidence from China
Source: Gerontologist. 2025 Apr 17;65(7):gnaf131. doi: 10.1093/geront/gnaf131 (PMC12257480; doi:10.1093/geront/gnaf131)
Supplement: gnaf131_suppl_Supplementary_Material [file gnaf131_suppl_supplementary_material.docx]

Appendix

Supplementary Figure 1 *Distribution of the LCCP programs in the three waves, 2010-2017*


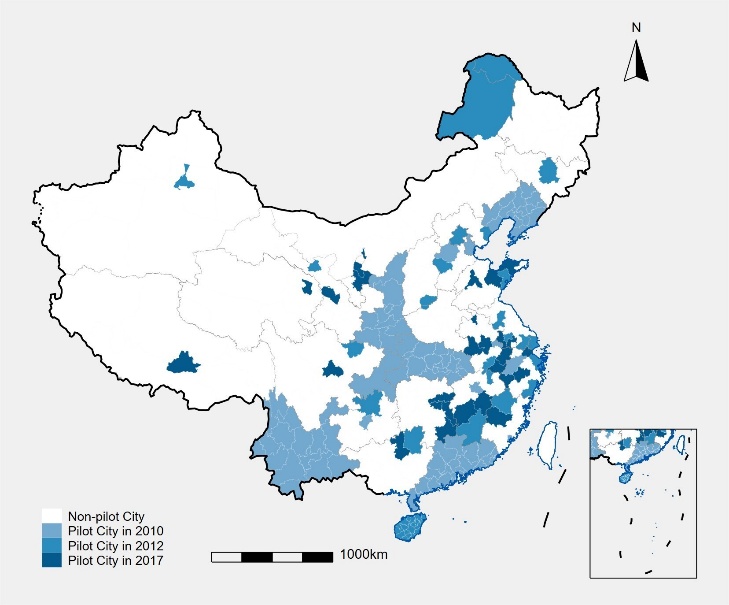


Alt text: A map of China showing the geographic distribution of pilot cities in the Low Carbon City Pilot (LCCP) program across three waves: 2010, 2012, and 2017. Cities are shaded in different tones by implementation year, while white areas represent non-pilot cities. The map indicates that LCCP coverage expanded substantially across eastern and central China over time.
